# Stress urinary incontinence after hysterectomy: a 10-year national follow-up study

**DOI:** 10.1007/s00404-021-06378-z

**Published:** 2022-01-21

**Authors:** Sari Tulokas, M. Mentula, P. Härkki, T. Brummer, J. Jalkanen, T. Kuittinen, J. Mäkinen, J. Sjöberg, E. Tomas, P. Rahkola-Soisalo

**Affiliations:** 1 University of Helsinki and Helsinki University Hospital, Helsinki, Finland; 2 Central Hospital Østfold, Fredrikstad, Norway; 3 Central Finland Hospital District, Jyvaskyla, Finland; 4 University of Turku, Turku, Finland; 5 Tampere University Hospital, Tampere, Finland

**Keywords:** Hysterectomy, Mid-urethral sling, Stress urinary incontinence, Urinary incontinence

## Abstract

**Purpose:**

Hysterectomy has been associated with increased risk for developing stress urinary incontinence (SUI) and having a SUI operation. We examined the long-term rate of SUI operations after hysterectomy and associated risk factors.

**Methods:**

We followed up 5000 women without prior urinary incontinence (UI) who had a hysterectomy in a prospective FINHYST 2006 cohort study until the end of 2016 through a national health register. The main outcome was SUI operations, and secondary outcomes were outpatient visits for UI, and their association of preoperative patient and operation factors.

**Results:**

During the median follow-up time of 10.6 years (IQR 10.3–10.8), 111 (2.2%) women had a SUI operation and 241 (4.8%) had an outpatient visit for UI. The SUI operation rate was higher after vaginal hysterectomy and laparoscopic hysterectomy (*n* = 71 and 28, 3.3% and 1.8%, respectively) compared to abdominal hysterectomy (*n* = 11, 0.8%). In a multivariate risk analysis by Cox regression, the association with vaginal hysterectomy and SUI operation remained significant when adjusted for vaginal deliveries, preceding pelvic organ prolapse (POP), uterus size, age and BMI (HR 2.4, 95% CI 1.1–5.3). Preceding POP, three or more deliveries and laparoscopic hysterectomy were significantly associated with UI visits but not with SUI operations.

**Conclusion:**

After hysterectomy, 2.2% of women underwent operative treatment for SUI. The number of SUI operations was more than double after vaginal hysterectomy compared to abdominal hysterectomy, but preceding POP explained this added risk partially. Preceding POP and three or more vaginal deliveries were independently associated with UI visits after hysterectomy.

## Introduction

Hysterectomy is a commonly used procedure to treat benign conditions such as abnormal uterine bleeding, pelvic organ prolapse (POP), and uterine fibroids. In the US alone, 433,621 inpatient hysterectomies were performed in 2010 [1]. The hysterectomy approach is decided mainly based on the indication and size of the uterus, but other patient-related factors and preferences of the gynecologic surgeon also affect the choice. Cochrane review recommends vaginal hysterectomy (VH) whenever possible, and in 2006 in Finland, VH was the most common approach, followed by laparoscopic hysterectomy (LH), which has largely replaced the abdominal hysterectomy (AH) [2, 3].

The way hysterectomy affects the pelvic anatomy and causes surgical trauma to the nerve supply is thought to cause the doubled risk of developing stress urinary incontinence (SUI) [4], defined as involuntary leakage of urine on effort or exertion [5]. Similarly, other factors that cause pressure or trauma to the pelvic floor, such as obesity, pregnancies and vaginal deliveries increase the risk of SUI [6]. However, the role of indication for hysterectomy, concomitant prolapse, concomitant surgery or the experience of the surgeon on the risk of SUI after hysterectomy remains unclear. The independent effect of the selected hysterectomy approach has also been hard to establish due to confounding factors [4].

In this prospective cohort study, we follow-up 5000 women for 10 years after a hysterectomy performed for a benign indication in 2006 and report the number of SUI operations and outpatient visits for SUI and other urinary incontinence (UI). We also assess the association of patient and operation factors with the long-term risk for SUI operations and UI visits.

## Materials and methods

A prospective national FINHYST 2006 study of 5279 women who underwent a hysterectomy for a benign indication was conducted in 53 Finnish hospitals in 2006, and it included 79% of all hysterectomies for benign indications in Finland that year. All women provided written informed consent before the study, and they also gave permission for further analyses. Data were collected in 2006 by surveys to gynecological surgeons and their patients as described in detail previously [2].

The follow-up data were extracted from the Care Register for Health Care (Care Register) maintained by the Finnish Institute for Health and Welfare. The Care Register contains diagnoses and operation codes for all in- and outpatient visits in every private and public hospital in Finland. The validity of the Care Register with respect to different medical conditions has been evaluated as satisfactory to very good in numerous studies, and a few validation studies have been published in the field of gynecology [7]. From the Care Register, we identified the sample women’s visits for urinary incontinence (ICD-10 code N39.3 for SUI, and N39.4 for other UI), visits for pelvic organ prolapse (ICD-10 code N81* and NCSP code LEF*) and SUI operations (NCSP codes LEG*, KDG*, KDV20 and KDV22) from 1996 to the end of 2016.

**Fig. 1:**
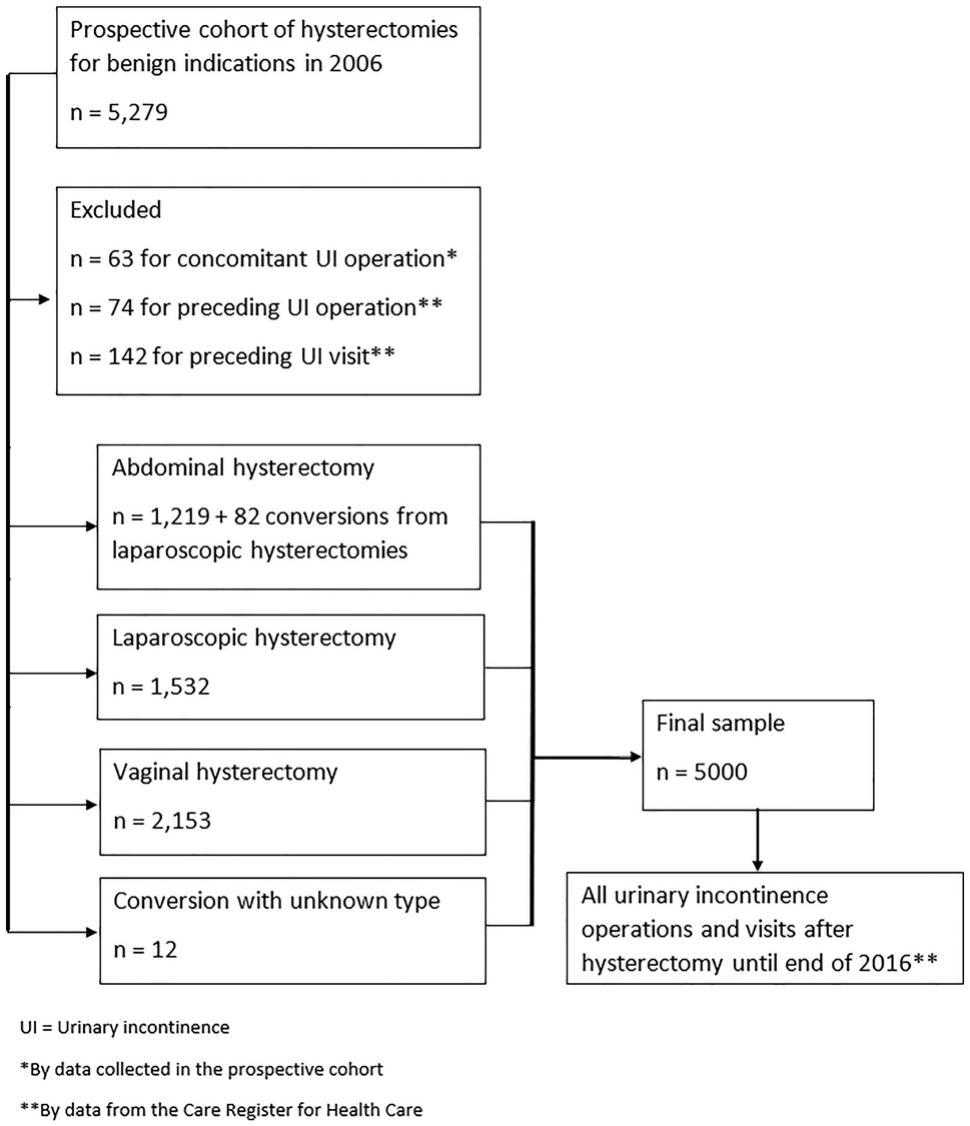
Flowchart of sample collecting process

The hysterectomy approaches were classified as vaginal, abdominal, and laparoscopic, and both total and subtotal hysterectomies were included. In the event of conversion, hysterectomy approach was classified according to the final operation method. We excluded women (*n* = 279) who had a concomitant UI operation recorded in the FINHYST 2006 data or visits with UI diagnosis or SUI operation codes (ICD-10 codes N39.3 and N39.4, NCSP codes LEG*, KDG*, KDV20, and KDV22) before or concomitant with the hysterectomy in the Care Register producing a final sample size of 5000 (Fig. 1).

Women were considered to have a preceding POP if the FINHYST 2006 survey data reported POP as the main indication or a concomitant POP operation, or if there was a hospital visit recorded in the Care Register before the hysterectomy with a diagnosis code for POP (ICD-10 N81*) or POP operation (NCSP code LEF*). As the indication, we used the single main preoperative indication selected by the gynecologic surgeon in the preoperative survey (myoma, menorrhagia, dysmenorrhea, endometriosis, uterine prolapse, adnexal mass or other). Other patient and operation characteristics were defined as described previously [2].

Our main outcome was SUI operation 60 days or more after the hysterectomy, which we defined as a visit with a SUI operation code (NCSP codes LEG*, KDG*, KDV20 and KDV22) in the Care Register. Our secondary outcome was UI visits 60 days or more after hysterectomy, which we defined as a visit with a UI diagnosis code (ICD-10 N39.3 for SUI, and N39.4 for other UI) in the Care Register. Only the first operation for SUI or visit for UI was reported for each woman. We defined UI visits that also had a diagnosis code for POP (ICD-10 N81*) as UI visits with concomitant POP.

In the risk factor analysis, we first conducted a univariate analysis for hysterectomy approach, age, BMI, preceding abdominal operations (including caesarean sections, laparoscopies, and laparotomies), preceding POP, parity, vaginal deliveries, indication, uterus size, concomitant operations, intraoperative complications, experience of the surgeon, and hospital type. We tested the variables that were found significant in the univariate analysis in a multivariate Cox regression analysis adjusted with each other. We conducted a similar risk analysis including only women without a preceding POP (*n* = 3452). For visualization, we made a Kaplan–Meier curves for cumulative survival without a SUI operation according to the hysterectomy method and preceding POP. When comparing more than two groups, we used one-way ANOVA to calculate the *p* value. A significance level of *p* < 0.05 was used unless stated otherwise. IBM SPSS Statistics 25 was used for statistical analysis.

The study protocol was approved by the Ethical Committee of the Helsinki and Uusimaa Hospital District (Dnro 457/E8/04 and 343/13/03/03/2015) and was registered in the Clinical Trials (NCT00744172). The Finnish Institute for Health and Welfare of Finland authorized the use of the data from the Care Register (THL/986/5.05.00/2018).

## Results

Of the 5000 hysterectomies, VH was most common, followed by LH and AH (Table 1). Subtotal procedures were rare: in AH 85 (6.5%) and in LH 3 (0.2%). Fibroids were the most common indication of all hysterectomies, followed by POP in the VH group. Concomitant surgery was performed in 2743 (55%) hysterectomies. Bilateral adnexal removal was performed for 36% AH and 32% in LH groups, while concomitant POP procedures were performed for 54% in the VH group.

**Table 1:** Sample demographics

	All hysterectomies^a^ (5000)	Abdominal hysterectomies (1301)	Laparoscopic hysterectomies (1532)	Vaginal hysterectomies (2153)
Age, median (IQR)	50 (45–57)	49 (45–54)	48 (44–53)	53 (46–63)
BMI, median (IQR)	26 (23–29)	26 (23–30)	25 (23–28)	26 (23–29)
Preceding abdominal operation^b^	2359 (47.2)	657 (50.5)	823 (53.7)	875 (40.6)
Preceding pelvic organ prolapse	1548 (31.0)	40 (3.1)	110 (7.2)	1394 (64.7)
Vaginal deliveries				
Nulliparous	861 (17.2)	377 (29.0)	339 (22.1)	144 (6.7)
Only caesarean sections	319 (6.4)	147 (11.3)	119 (7.8)	52 (2.4)
1 or 2	2440 (48.8)	553 (42.5)	741 (48.4)	1138 (52.9)
3 or more	1380 (27.6)	224 (17.2)	333 (21.7)	819 (38.0)
Main indication				
Pelvic organ prolapse	1346 (26.9)	1 (0.1)	47 (3.1)	1296 (60.2)
Fibroids	1687 (33.7)	759 (58.3)	584 (38.1)	336 (15.6)
Dysfunctional uterine bleeding	1047 (20.9)	194 (14.9)	457 (29.8)	393 (18.3)
Dysmenorrhea	135 (2.7)	25 (1.9)	67 (4.4)	42 (2.0)
Endometriosis	129 (2.6)	84 (6.5)	42 (2.7)	3 (0.1)
Other	656 (13.1)	238 (18.3)	335 (21.9)	83 (3.9)
Uterus size, median in grams (IQR)	150 (87–275)	309 (150–550)	170 (110–259)	101 (59–160)
Any concomitant operation	2743 (54.9)	735 (56.5)	755 (49.3)	1250 (58.1)
Any concomitant POP operation	1246 (24.9)	15 (1.2)	78 (5.1)	1151 (53.5)
Concomitant anterior colporrhaphy	1043 (20.9)	4 (0.3)	51 (3.3)	987 (45.8)
Concomitant posterior colporrhaphy	757 (15.1)	10 (0.8)	46 (3.0)	700 (32.5)
Concomitant other POP operation	29 (0.6)	1 (0.1)	0 (0)	28 (1.3)
Bilateral adnexal removal	1002 (20.0)	464 (35.7)	492 (32.1)	46 (2.1)
Experience of surgeon				
< 10 hysterectomies	528 (10.6)	130 (10.0)	193 (12.6)	205 (9.5)
10–30 hysterectomies	549 (11.0)	153 (11.8)	153 (10.0)	242 (11.2)
Over 30 hysterectomies	3738 (74.))	975 (74.9)	1125 (73.4)	1626 (75.5)
Not available	185 (3.7)	43 (3.3)	61 (4.0)	80 (3.7)
Specialist as surgeon	3619 (72.4)	950 (73.0)	1177 (76.8)	1480 (68.7)
Resident as surgeon	1092 (21.8)	283 (21.8)	263 (17.2)	545 (25.3)
Status of surgeon not available	289 (5.8)	68 (5.2)	92 (6.0)	128 (5.9)
Operation in university hospital	1848 (37.0)	392 (30.1)	779 (50.8)	674 (31.3)
Operation in central hospital	1929 (38.6)	614 (47.2)	461 (30.1)	848 (39.4)
Operation in local hospital	1176 (23.5)	289 (22.2)	267 (17.4)	615 (28.6)
Operation in private hospital	47 (0.9)	6 (0.5)	25 (1.6)	16 (0.7)
Any intraoperative complication	726 (14.5)	261 (20.1)	215 (14.0)	245 (11.4)
Minor intraoperative complication	580 (11.6)	211 (16.2)	167 (10.9)	198 (9.2)
Major intraoperative complication	174 (3.5)	64 (4.9)	53 (3.5)	56 (2.6)

*IQR* interquartile range, *BMI* body mass index, *POP* pelvic organ prolapse

^a^Including abdominal, laparoscopic, vaginal hysterectomies and 12 converted hysterectomies with unknown final operation method

^b^Including caesarean sections, laparoscopies and laparotomies

During the follow-up time of 10.6 median years (IQR 10.3–10.8), 111 (2.2%) women had a SUI operation (Table 2). The rate of SUI operations was significantly higher after VH (*n* = 71, 3.3%) than after AH and LH (*n* = 11, 0.8% and *n* = 28, 1.8%, respectively, *p* < 0.05). The mid-urethral sling was the predominant operation type (97%), and the transobturator route was slightly more frequently used than retropubic route (53% and 44%, respectively). The operations were mostly performed due to SUI (*n* = 96, 87%). Of all women, 241 (4.8%) had a hospital visit for urinary incontinence, of which 142 (59%) were for SUI.

**Table 2:** Stress urinary incontinence operations and urinary incontinence visits after hysterectomy

	All hysterectomies (5000)	Abdominal hysterectomies (1301)	Laparoscopic hysterectomies (1532)	Vaginal hysterectomies (2153)	*p*
Any SUI operation (*n*, %)	111 (2.2)	11 (0.8)	28 (1.8)	71 (3.3)	< 0.05*
RP-MUS	49 (44.1)	5 (45.5)	16 (57.1)	27 (38.0)	
TO-MUS	59 (53.2)	6 (54.5)	12 (42.9)	41 (57.7)	
Bulking injection	0	0	0	3 (4.2)	
Other	3 (2.7)	0	0	0	
Indication for incontinence operation (*n*, %)					
Stress urinary incontinence	96 (86.5)	10 (90.9)	27 (96.4)	58 (81.7)	
Mixed urinary incontinence	15 (13.5)	1 (9.1)	1 (3.6)	13 (18.3)	
Time to operation, median in years (IQR)	3.8 (2.9–7.2)	3.2 (2.0–6.7)	4.6 (2.9–8.2)	3.2 (1.7–6.4)	0.3
Any incontinence visit (*n*, %)	241 (4.8)	32 (2.5)	68 (4.4)	139 (6.5)	< 0.05*
Stress urinary incontinence visit	142 (58.9)	18 (56.3)	42 (61.8)	80 (57.6)	
Other urinary incontinence visit	99 (41.1)	14 (43.8)	26 (38.2)	59 (42.4)	
Time to re-visit, median in years (IQR)	3.4 (1.2–6.5)	3.6 (1.5–6.8)	3.9 (1.9–6.9)	2.8 (0.7–6.3)	0.3

*SUI* stress urinary incontinence, *RP-MUS* retropubic mid-urethral sling, *TO-MUS* transobturator mid-urethral sling, *POP* pelvic organ prolapse, *IQR* interquartile range

*Statistically significant

In the univariate analysis, the SUI operations and UI visits were significantly associated with LH and VH when AH was used as the reference (Table 3). Preceding POP and POP as the main indication were equally associated with SUI operation and UI visit, while other indications were insignificant. In a sub-analysis including only patients with a preceding POP (*n* = 1548), a concomitant POP operation did not affect the risk for SUI operation or UI visit (HR 1.0 and 0.9, 95% CI 0.5–2.0 and 0.6–1.3, respectively). Age, BMI, experience or status of the surgeon, hospital type, preceding abdominal operations, concomitant adnexal removal, any concomitant operation, and perioperative complications (any, minor, or major) were found statistically insignificant in univariate analysis (*p* > 0.2).

**Table 3:** Univariate risk analysis for SUI operation and UI visits after hysterectomy

Univariate analysis	SUI operation	UI visit
	HR (95% CI)	HR (95% CI)
Age (continuous)	1.0 (1.0–1.0)	1.0 (1.0–1.0)
Body mass index (continuous)	1.0 (1.0–1.0)	1.0 (1.0–1.0)
Hysterectomy approach		
Abdominal	(reference)	(reference)
Laparoscopic	2.2 (1.1–4.4)*	1.8 (1.2–2.8)*
Vaginal	4.0 (2.1–7.5)*	2.7 (1.8–3.9)*
Main indication		
Fibroids	(reference)	(reference)
Pelvic organ prolapse	2.0 (1.2–3.3)*	2.3 (1.7–3.1)*
Dysfunctional uterine bleeding	0.9 (0.5–1.6)	1.3 (0.8–1.9)
Dysmenorrhea	1.3 (0.4–4.2)	1.3 (0.6–3.0)
Endometriosis	0.9 (0.2–3.7)	0.9 (0.3–2.5)
Other	1.2 (0.7–2.4)	1.1 (0.7–1.8)
Uterus size (continuous in 100 g steps)	0.9 (0.8–1.0)*	0.8 (0.8–0.9)*
Uterus size categorial		
< 300	(reference)	(reference)
300–500	0.6 (0.3–1.2)	0.5 (0.3–0.9)*
Higher than 500	0.2 (0.04–0.7)*	0.3 (0.2–0.7)*
Concomitant operations		
Any concomitant operation	1.4 (0.9–2.0)	1.6 (1.2–20)*
Any POP operation	1.9 (1.3–2.7)*	2.0 (1.6–2.6)*
Anterior colporrhaphy	1.8 (1.2–2.7)*	1.8 (1.4–2.3)*
Posterior colporrhaphy	2.1 (1.4–3.2)*	2.2 (1.7–2.9)*
Other POP operation	3.3 (0.8–13.2)	1.5 (0.4–5.9)
Bilateral adnexal removal	0.5 (0.3–0.9)*	0.7 (0.5–1.0)*
Parity		
Nulliparous	(reference)	(reference)
1 or 2	1.5 (0.8–2.8)	1.3 (0.9–2.0)
3 or more	2.2 (1.1–4.1)	2.0 (1.3–3.1)*
Vaginal deliveries		
None	(reference)	(reference)
1 or 2	2.9 (1.0–3.4)*	1.5 (1.0–2.1)*
3 or more	2.7 (1.5–4.9)*	2.4 (1.6–3.4)*
Preceding POP	2.0 (1.4–2.9)*	2.3 (1.8–3.0)*

*SUI* stress urinary incontinence, *UI* urinary incontinence, *POP* pelvic organ prolapse

*Statistically significant

In multivariate analysis, only vaginal hysterectomy was associated with a significantly higher risk for SUI operations (HR 2.4, 95% CI 1.1–5.3, see Table 4). Patients with a higher number of vaginal deliveries, smaller uterus size and preceding POP did have more SUI operations, but this difference did not reach statistical significance. As to UI visits, a significant association was found with preceding POP (HR 2.0, 95% CI 1.4–2.9), three or more vaginal deliveries (HR 1.7, 95% CI 1.1–2.5), and LH (HR 1.6, 95% CI 1.0–2.6). Preceding POP was significantly associated with other UI visits but not with SUI visits (HR 3.1 and 1.5, 95% CI 1.8–5.6 and 0.9–2.3, respectively).

**Table 4:** Multivariable risk analysis for SUI operations and UI visits after hysterectomy

All sample women included	SUI operation	UI visit	SUI visit	MUI visit
All factors adjusted with each other	HR (95% CI)	HR (95% CI)	HR (95% CI)	HR (95% CI)
Hysterectomy approach				
Abdominal	(reference)	(reference)	(reference)	(reference)
Laparoscopic	1.7 (0.8–3.7)	1.6 (1.0–2.6)*	1.6 (0.9–3.0)	1.6 (0.8–3.4)
Vaginal	2.4 (1.1–5.3)*	1.3 (0.8–2.3)	1.6 (0.8–3.2)	1.0 (0.4–2.3)
Vaginal deliveries				
None	(reference)	(reference)	(reference)	(reference)
1 or 2	1.3 (0.7–2.5)	1.1 (0.7–1.7)	1.3 (0.8–2.2)	0.9 (0.5–1.7)
3 or more	1.8 (0.9–3.5)	1.7 (1.1–2.5)*	1.5 (0.9–2.7)	1.8 (0.9–3.3)
Uterus size				
< 300 g	(reference)	(reference)	(reference)	(reference)
300 g to < 500 g	0.8 (0.4–1.7)	0.7 (0.4–1.2)	0.6 (0.3–1.2)	0.9 (0.4–2.0)
500 g or larger	0.3 (0.1–1.4)	0.6 (0.3–1.3)	0.4 (0.2–1.3)	1.0 (0.3–2.7)
Preceding POP (ref: no preceding POP)	1.2 (0.7–1.9)	2.0 (1.4–2.9)*	1.5 (0.9–2.3)	3.1 (1.8–5.6)*
Concomitant adnexal removal (ref: no removal)	0.8 (0.4–1.6)	0.9 (0.6–1.3)	1.1 (0.6–1.8)	0.6 (0.3–1.2)

Only women without preceding POP included	SUI operation	UI visit	SUI visit	MUI visit
All factors adjusted with each other	HR (95% CI)	HR (95% CI)	HR (95% CI)	HR (95% CI)
Vaginal deliveries				
No vaginal deliveries	(ref)	(ref)	(ref)	(ref)
1–2	1.5 (0.7–3.0)	1.2 (0.7–1.9)	1.3 (0.7–2.3)	1.0 (0.4–2.2)
3 or more	1.4 (0.6–3.2)	1.8 (1.1–3.0)*	1.6 (0.8–3.1)	2.2 (1.0–4.9)*
Hysterectomy approach				
Abdominal	(ref)	(ref)	(ref)	(ref)
Laparoscopic	1.7 (0.8–3.6)	1.4 (0.8–2.2)	1.5 (0.8–2.9)	1.1 (0.5–2.4)
Vaginal	2.5 (1.1–5.9)*	1.3 (0.7–2.3)	1.6 (0.8–3.4)	0.8 (0.3–2.1)
Uterus size				
< 300 g	(ref)	(ref)	(ref)	(ref)
300 g to < 500 g	1.0 (0.5–2.0)	0.7 (0.4–1.3)	0.7 (0.4–1.5)	0.8 (0.3–1.9)
500 g or larger	0.3 (0.1–1.5)	0.5 (0.2–1.1)	0.5 (0.2–1.4)	0.5 (0.1–1.8)
Concomitant adnexal removal (ref: no removal)	0.9 (0.5–1.9)	1.0 (0.6–1.5)	1.2 (0.7–2.1)	0.7 (0.3–1.5)

*SUI* stress urinary incontinence, *UI* urinary incontinence, *POP* pelvic organ prolapse

*Statistically significant

In the univariate sub-analysis including only women without preceding POP, LH and VH as the hysterectomy method were found significantly associated with both SUI operations (HR 2.0 and 3.4, 95% CI 1.0 and 4.1, respectively) and UI visits (HR 1.5 and 1.6, 95% CI 1.0–2.4 and 1.0–2.6, respectively). Uterus larger than 500 g was found negatively associated with SUI operations and visits (HR 0.2 and 0.4, 95% CI 0.1–0.9 and 0.2–0.9, respectively). Three or more vaginal deliveries were found significantly associated with UI visits but not with SUI operations (HR 1.8 and 1.4, 95% CI 1.1–3.0 and 0.7–3.1) when no vaginal deliveries was used as the reference. One to two vaginal deliveries, age, BMI, preceding abdominal operations, vaginal deliveries, main indication, concomitant procedures, experience and status of the surgeon and hospital type were found insignificant. In the multivariate sub-analysis including only women without preceding POP, VH remained significantly associated with SUI operations (HR 2.6, 95% CI 1.2–5.7) and three or more vaginal deliveries with UI visits (HR 1.8, 95% CI 1.1–3.0). See details of the multivariate analysis in Table 4.

**Fig. 2:**
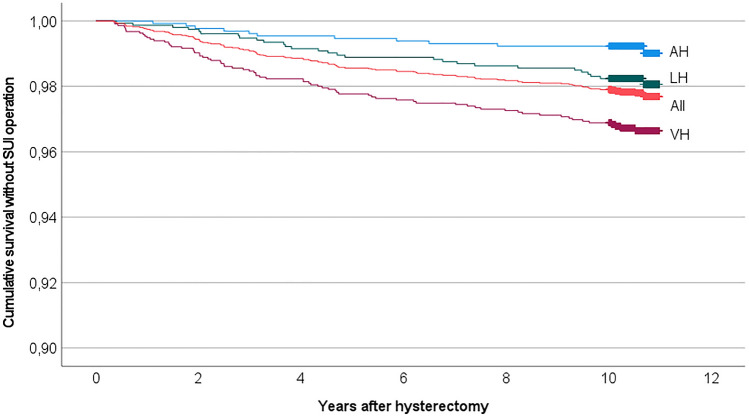
Kaplan–Meier cumulative survival without SUI operation after hysterectomy according to hysterectomy approach

**Fig. 3:**
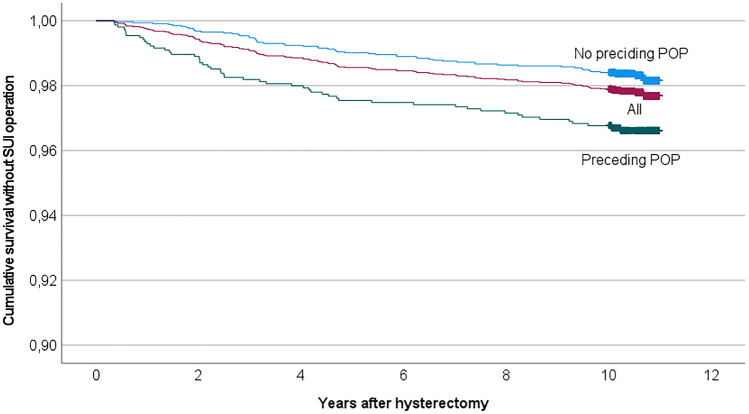
Kaplan–Meier cumulative survival without SUI operation after hysterectomy with or without preceding POP

Kaplan–Meier curves of survival without SUI operation (Figs. 2, 3) show that SUI operations occurred throughout the follow-up time. However, most UI visits (59%) and SUI operations (57%) occurred within the first 5 years after the hysterectomy. The difference in the cumulative survival between the different hysterectomy approaches and women with or without preceding POP lasts throughout the follow-up time. The median time from hysterectomy to SUI operations and UI visits did not differ significantly between the different hysterectomy approaches (*p* = 0.3). The median time between hysterectomy and UI visit was significantly shorter for women with preceding POP compared to women without preceding POP (median time in years 2.6 and 4.3, IQR 0.6–6.5 and 1.7–6.6, respectively, *p* < 0.05).

## Discussion

In this prospective cohort study of 5000 hysterectomies on women without prior UI, we assessed the risk for SUI operation after hysterectomy. The long follow-up time of over 10 years ensured that our study most likely could record all new UI cases since our data are similar to the previous study by Altman et al., with the risk being highest up to 5 years after hysterectomy [4]. Even though only 2.2% of women had a SUI operation 10 years after hysterectomy, the rate of SUI operations was twice higher than expected based on national incidence of SUI operations in Finland in 2009 [8]. The rate of SUI operations was approximately half of the rate of UI visits (2.2% vs 4.8%), which indicates that some of the women seeking help managed with conservative treatments.

We found the risk for SUI operation to be over twice higher after VH than AH even after adjusting for other risk factors and when only women without preceding POP were included in the analysis. This is in line with a previous large cohort study in Sweden [9]. However, because the choice of hysterectomy method was not randomized, it is possible that the women in the VH group had favorable anatomy for VH such as mobile uterus or latent POP.

Differences of re-establishing apical support between hysterectomy approaches may also explain the added risk for SUI operations and UI visits after LH compared to AH. At the time of the sample hysterectomies, uterosacral ligament fixation was not used in LH, which may explain the significantly higher risk for UI visits after LH compared to AH. Re-establishing apical support at hysterectomy is recommended to prevent post-hysterectomy POP [10], but its effect on de novo UI after hysterectomy is not established, and in the future it will be interesting to see whether laparoscopic suturing of the vaginal cuff with uterosacral ligament fixation will decrease the risk of UI. However, because uterosacral ligament fixation is used in VH, the differences in building apical support does not explain the risk associated with VH. It is possible that at least some women in VH group had latent POP, and dysfunction of pelvic floor resulted also in UI. Also, most women with uterus larger than 500 g had AH. It is possible that a uterus too large to prolapse may protect structures from stretching down, and thus, decrease the risk for UI after AH. Even though the protective effect of a uterus larger than 500 g was no longer significant in our multivariate analysis, previously, a uterus larger than 500 g has been associated with a lower risk for UI after hysterectomy [11].

Preceding POP significantly increased the risk for UI visit but not for SUI operation when adjusted for other risk factors. This result is supported by a previous study that reported an unadjusted HR of 1.7 for SUI operation after hysterectomy with POP as indication [4]. In our data, preceding POP had a stronger association with visits for other UI than with visits for SUI. It is likely that many of these women suffered from urge dominant UI, which is treated conservatively. Therefore, this finding could explain why the risk for SUI operation remained insignificant. Interestingly, women with a concomitant POP operation had a similar rate of SUI operations and UI visits than women with a preceding POP without a concomitant POP operation. Association between SUI and POP has been noted previously, and after a POP operation without hysterectomy, up to 39% of women without SUI and 52% of women with occult SUI preoperatively present with objective SUI [12]. These data reflect that both SUI and POP are a result of pelvic floor dysfunction and a complex collection of interconnected symptoms.

Even though bilateral adnexal removal can also be performed in VH, it is often easier to perform in AH or LH. Unsurprisingly, only 5% of hysterectomies in our sample with a concomitant bilateral adnexal removal were performed in VH. This uneven distribution between hysterectomy approaches explains why bilateral adnexal removal was found significantly associated with SUI operations and UI visits only in the univariate analysis but not in the multivariate analysis.

The strengths of this study include a large prospective sample of 5000 women, unselected population-based design that represents real-life clinical setting, long follow-up time of 10.6 years in median and clear end-point data from a reliable register. The detailed patient and operation characteristics data collected preoperatively allowed us to control confounding factors such as BMI, age, preceding POP, vaginal deliveries, uterus size, and concomitant deliveries. Furthermore, being able to distinguish SUI visits from visits for other UI allowed us to analyze risk factors for pure SUI without confounding other types of UI.

As a limitation, we recognize that we were not able to identify UI in women who did not seek treatment or were not referred to specialized health care. However, we believe that the operations and visits represent well bothersome UI due to good access to healthcare in Finland. Using SUI operations and UI visits as endpoints also resulted in a low number of end-point events and lower statistical power despite the large sample size. Because the hysterectomy method was not chosen randomly, there were many demographic differences between AH, LH and VH groups. To overcome this selection bias, we used multivariable analysis and performed sub-group analysis when needed, but we acknowledge that not all potentially significant confounders such as uterus mobility were captured in the data. Even though BMI is a known risk factor for SUI, in our sample it was not significantly associated with SUI operations or visits. This result may be biased, because obesity is a relative contraindication for surgical treatment of UI as well as hysterectomy. Also, even though we had detailed information collected at the time of the hysterectomy, the profession, menopausal status, comorbidities and home medications were not included in our data.

To conclude, during the follow-up time of ten years, 2.2% of women had a SUI operation after hysterectomy. The association of VH and SUI operations remained significant even after adjusting for other risk factors and in a sub-analysis including only women without preceding POP. Preceding POP was significantly associated with UI visits, and this association was stronger with other UI visits than SUI visits. Also, three or more vaginal deliveries and LH were significantly associated with UI visits.

## Data Availability

The data and material are not available.

## Code availability

IBM SPSS Statistics 25 was used for statistical analysis.
